# TOSD: A Hierarchical Object-Centric Descriptor Integrating Shape, Color, and Topology

**DOI:** 10.3390/s25154614

**Published:** 2025-07-25

**Authors:** Jun-Hyeon Choi, Jeong-Won Pyo, Ye-Chan An, Tae-Yong Kuc

**Affiliations:** 1 Department of Electrical and Computer Engineering, College of Information and Communication Engineering, Sungkyunkwan University, Suwon 16419, Republic of Korea; choijunhyeon@g.skku.edu (J.-H.C.); ycan@g.skku.edu (Y.-C.A.); 2 R&D Center, DXR Co., Ltd., Seoul 01411, Republic of Korea; jungwon900@dxr-ai.com

**Keywords:** hierarchical descriptor, visual representation, scene understanding, object pooling, feature aggregation

## Abstract

This paper introduces a hierarchical object-centric descriptor framework called TOSD (Triplet Object-Centric Semantic Descriptor). The goal of this method is to overcome the limitations of existing pixel-based and global feature embedding approaches. To this end, the framework adopts a hierarchical representation that is explicitly designed for multi-level reasoning. TOSD combines shape, color, and topological information without depending on predefined class labels. The shape descriptor captures the geometric configuration of each object. The color descriptor focuses on internal appearance by extracting normalized color features. The topology descriptor models the spatial and semantic relationships between objects in a scene. These components are integrated at both object and scene levels to produce compact and consistent embeddings. The resulting representation covers three levels of abstraction: low-level pixel details, mid-level object features, and high-level semantic structure. This hierarchical organization makes it possible to represent both local cues and global context in a unified form. We evaluate the proposed method on multiple vision tasks. The results show that TOSD performs competitively compared to baseline methods, while maintaining robustness in challenging cases such as occlusion and viewpoint changes. The framework is applicable to visual odometry, SLAM, object tracking, global localization, scene clustering, and image retrieval. In addition, this work extends our previous research on the *Semantic Modeling Framework*, which represents environments using layered structures of places, objects, and their ontological relations.

## 1. Introduction

Visual representation techniques have advanced significantly in recent years, leading to better performance in tasks such as object detection, segmentation, and retrieval, even in complex scenes. Despite these improvements, many conventional approaches still rely heavily on low-level features, such as keypoints, edges, or pixel patterns. These methods often struggle to capture high-level semantics, such as spatial structure and inter-object relationships, which limits their ability to generalize and interpret real-world environments. They are susceptible to occlusions, viewpoint changes, and variations in object appearance.

Recent studies on multimodal feature fusion [1,2] show that integrating complementary visual features improves both semantic understanding and robustness. Inspired by this, we design a hierarchical representation framework that combines information across three levels: low-level pixel features, mid-level attributes such as shape and color, and high-level semantics that describe object relationships.

To address the shortcomings of low-level representations, we present TOSD (Triplet Object-Centric Semantic Descriptor), which describes an image using object-centric cues based on shape, color, and topology. TOSM combines heterogeneous visual features to represent both local appearance and global structure. This results in robust and discriminative embeddings that support a wide range of tasks, including object pooling, image matching, and scene retrieval. The framework also extends to downstream applications such as visual odometry, SLAM, object tracking, global localization, and scene-level clustering.

Our work builds on the Semantic Modeling Framework [3], which organizes spatial environments into layered structures involving coarse-to-fine places, object instances, and semantic associations. This framework has already been applied to various domains, including multi-robot systems [4,5] and autonomous vehicles [6].

For object extraction, we rely on off-the-shelf segmentation models and salient point detectors. In particular, we use a zero-shot segmentation model [7,8] that performs well even without task-specific training. This approach enables the system to handle unknown or semantically minor objects, which may still play an important role in understanding the scene.

The main contributions of this work are as follows:

1. We propose TOSD, a unified object-centric descriptor framework that jointly encodes shape, color, and topology for robust visual matching.
2. We introduce a hierarchical representation strategy that extends from low-level pixel information to mid-level object semantics and high-level scene understanding.
3. Encodes shape, color, and scene information robustly across varying views using relation-based encoding, while attention-based filtering and fusion enhance efficiency by removing irrelevant relations.
4. Supports various vision tasks and provides a foundation for the Semantic Modeling Framework, which defines the environment by hierarchical representation.

## 2. Related Works

### 2.1. Keypoint Descriptors and Dense Matching

Traditional local feature descriptors such as SIFT [9] and SURF [10] have been widely used for keypoint-based feature matching [11]. These methods are generally robust to changes in scale and rotation but remain sensitive to variations in illumination and viewpoint.

Recent deep learning-based methods, including SuperPoint [12], D2-Net [13], and R2D2 [14], offer improved keypoint stability and discriminative power. Although these approaches enhance matching performance, they are fundamentally limited by their reliance on local structures, which makes it difficult to incorporate global context or object-level semantic information. Dense matching, such as LoFTR and Patch2Pix, attempts to overcome keypoint sparsity by establishing pixel-wise correspondences [15,16].

However, dense matching causes significant computational overhead and often ignores object-level semantics. The methods mentioned above have significantly improved image correspondence performance. However, most of these rely on local information and cannot capture semantic relationships in images. To address these limitations, we describe image pixel object scene level hierarchical representations, aiming to capture object-centric structural features and relational awareness.

### 2.2. Object-Centric Representation and Pooling

Object-centric representations rise for supporting structured reasoning and compositional generalization [17,18]. Slot Attention and Object-Centric Transformers represent scenes as sets of independent objects with learnable embeddings [19,20]. These methods typically train in unsupervised or weakly supervised strategy to discover meaningful object-level structures. Object scene representation, such as OSRT, has demonstrated that representing scenes at the object level enables learning more structured and interpretable embeddings. By integrating object-based inductive biases, these methods enhance representation quality and semantic consistency, while object pooling contributes to global information integration and improved generalization [21,22].

However, many existing object-centric approaches tend to rely, to some extent, on explicit class information or prior knowledge, or focus primarily on learning implicit structural representations. This work explores an alternative direction by constructing object representations that integrate shape, color, and topology without requiring class supervision.

### 2.3. Multimodality Feature Extraction and Fusion

Multimodal and multiscale feature fusion has become increasingly important in visual perception tasks. By combining diverse sensor or signal inputs, models can build more comprehensive and stable representations.

Typical modalities used for fusion include RGB images (for texture and color), depth maps (for 3D geometry) [23], thermal [24] or infrared imagery (for heat-related cues) [25], optical flow (for motion tracking) [26,27], and frequency-domain features that capture periodicity [28] or structural patterns [29]. In addition, panoramic images [1] and stereo/multi-view videos [30] provide a wide field of view or geometric depth via parallax.

These multimodal sources are particularly useful in robotic navigation, environmental perception, and immersive scene reconstruction. Since each modality offers a different view of the environment, their fusion can reduce ambiguity or noise inherent in individual inputs. For example, while RGB images may suffer from occlusion, depth or thermal signals can still provide reliable information.

Recent advances in multimodal representation learning have proposed various architectural strategies to integrate diverse features across spatial and semantic levels [2]. Among these, multiscale attention mechanisms [31], hierarchical encoder-decoder frameworks [32], and residual fusion modules across modalities [33] have shown notable success in capturing both local appearance and global structure. Transformer-based encoders [34], for instance, employ multi-head self-attention across spatial hierarchies, enabling the model to incorporate long-range dependencies and contextual alignment. In contrast, convolutional architectures typically rely on dilated convolutions and residual blocks to extract multi-resolution features while preserving spatial detail [35]. In practical applications such as industrial inspection, medical diagnostics, and scene analysis, frequency-aware fusion techniques designed to emphasize structural regularities or subtle surface patterns have also demonstrated high utility [36]. These methods typically operate at the pixel or region level and assume the availability of synchronized multimodal inputs, such as RGB, depth, or thermal imagery. Despite their effectiveness, most of these approaches focus on fusing mid-level representations without explicitly modeling object-level structure or semantic relations.

This strategy has been widely adopted in domains requiring robust perception, including medical imaging [37], automated defect detection [36], scene understanding for robotics [38], and environmental sensing in autonomous vehicles [39].

In contrast, our TOSD framework performs semantic-level fusion of structurally heterogeneous features-shape, color, and topological relations-extracted from a single RGB image. While not multimodal in the traditional cross-sensor sense, the integration of these cues mimics multimodal fusion in terms of abstraction and architectural complexity. This object-level embedding strategy enables relational reasoning and scene generalization across tasks such as image matching, object retrieval, and visual odometry.

### 2.4. Graph-Based Feature Representation

Graph-based methods have long been used to represent relational information in scenes [40,41]. Scene graph generation models are capable of explicitly capturing relationships such as spatial arrangements and object interactions. [42] These structured representations have been utilized in various image-based tasks, enabling more context-aware and fine-grained reasoning [43,44]. Graph neural networks (GNNs) such as GCN [45], GAT [46], and GINE [47] are widely adopted for propagating context and learning object interactions. More recent hybrid models combine GNNs with transformers to exploit both local and global context [48,49]. In addition, GNNs have gained attention as an approach that integrates information through a combination with various network architectures, enabling both expressive power and generalization capability [50,51].

Although GNN-based methods rely on explicit relational structures and are sensitive to graph quality, they have emerged as a flexible framework capable of integrating local and global context through combination with various network architectures. In this study, we explore an effective representation approach that addresses these limitations.

### 2.5. Global Embedding for Retrieval and Matching

Embedding-based methods like NetVLAD and DELF have shown strong performance in retrieval tasks [52,53].These methods compress global or regional features into a high-dimensional embedding space, offering advantages in computational efficiency and global comparison. However, they have limitations in explicitly representing object boundaries, relationships, and compositional structures. Accordingly, recent approaches have focused on enhancing object-level representation and incorporating structural relationships and hierarchical semantics within a scene. R-MAC [54] generates global representations by aggregating region-based features, while TransVPR [55] leverages a transformer architecture to integrate contextual information between global and local levels. DINOv2 [56] enables precise retrieval and matching by jointly learning semantic clustering and hierarchical representations through large-scale pretraining.

While embedding-based approaches offer advantages in efficiency and global comparison, they are limited in effectively capturing structural relationships and the semantic organization of objects. Accordingly, for more fine-grained scene understanding, representations that encode object-centric structure and relational context are required.

In this paper, we present a hierarchical descriptor that addresses the limitations of existing pixel-level and global embedding methods. The proposed approach integrates object-level shape, color, and topological information without depending on predefined class labels. The descriptor, referred to as TOSD, is specifically designed to capture object-level relationships and compositional structures explicitly.

By combining various representation strategies within a graph neural network (GNN) framework, TOSD enables hierarchical integration of both local and global contextual cues. This design allows for flexible interpretation of visual scenes, supporting robust and compositional scene understanding.

## 3. Method

### 3.1. Overview of TOSD Architecture

**Figure 1:**
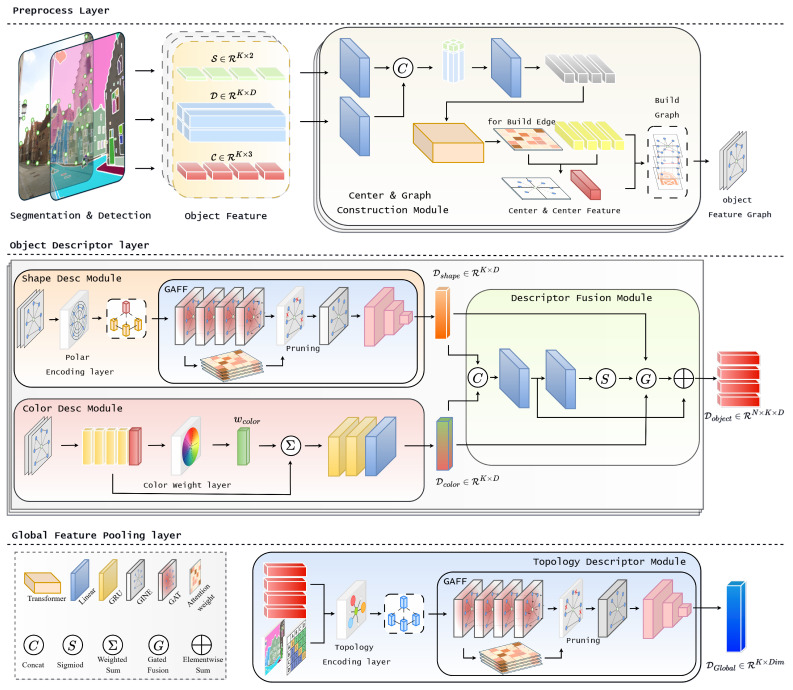
Overview of the proposed network architecture.

TOSD, which models images as object-centric hierarchical representations to enable image interpretation from various perspectives. As shown in Figure 1, TOSD extracts segmentation of the input image using a zero-shot segmentation model [7,8]. By casting a zero-shot approach, the framework can extract object features without predefined class information that are topologically meaningful elements, such as architectural boundaries and functional structures. This zero-shot approach offers the advantage of flexibly integrating various semantically meaningful regions without requiring task-specific supervision.

For each segmented object, sparse salient point detectors [9,12] are used to extract local features. The relationships among these features are quantified and represented through edge information defined in terms of shape, color, and topology, thereby effectively modeling the structural and semantic properties of the object.

Image is hierarchically represented through three complementary types of descriptors:

1. Shape Descriptor that represents geometric structure.
2. Color Descriptor that represents visual appearance.
3. Topology Descriptor that represents inter-object relationships.

Triplet descriptor performs jointly to describe an object by its intrinsic properties and contextual placement. This hierarchical structure of aggregated representations enables various perspective interpretations of complex scenes and provides a flexible foundation for various vision tasks. TOSD adopt off-the-shelf modules (e.g., SAM and SuperPoint), ensuring modularity, ease of integration, and compatibility with future methodological improvements.

### 3.2. Descriptor Modules

To effectively represent objects in a scene, we design three complementary descriptor modules, each focusing on a different aspect of the image. These modules are specialized for geometry, appearance, and inter-object structure, enabling TOSD to integrate diverse information into a unified object-centric embedding.

The shape descriptor is responsible for modeling the geometric properties of each object by utilizing the configuration of salient points. Through the use of spatial relationships and attention mechanisms, this module captures the overall contour and structural form of the object.

The color descriptor focuses on visual appearance by extracting normalized color information within the object region. It emphasizes internal color distribution, which can be critical in cases where geometric cues are limited or unclear.

The topology encoder models semantic and spatial relationships between objects by constructing a graph over the entire scene. This module captures contextual information, such as relative position, proximity, and semantic similarity, extending beyond the boundaries of individual objects.

In the following subsections, we describe the architecture and implementation details of each module.

#### 3.2.1. Center Pooling and Graph Construction

To integrate local features of an object efficiently, it is effective to model their internal relationships and construct corresponding representations [57,58]. However, when the number of features increases, such methods can suffer from high computational cost. To mitigate this, we construct a directed graph in which the object center serves as the root node, allowing the representation to be more structured and computation to be more efficient.

By using the object center as a reference, feature relationships can be organized based on both structural layout and semantic hierarchy. The directed edges enable controlled information flow, improving representational compactness and reducing redundancy during message passing. For this structure to be stable, the accuracy of the center is important. Therefore, we employ a Transformer-based module to evaluate the importance of each salient point using attention and compute a weighted centroid accordingly.

This estimated center remains semantically stable even in cases where local features are partially missing or unreliable due to shape deformation, viewpoint change, or occlusion. It acts as a global anchor that helps normalize the spatial arrangement and preserves the geometric and color-related consistency of the object.

The constructed graph consists of two types of connections:

1. Directed edges from the object center to salient points, forming a hierarchical structure.
2. Relevance-based connections between salient points to capture contextual or semantic relationships.

**Figure 2:**
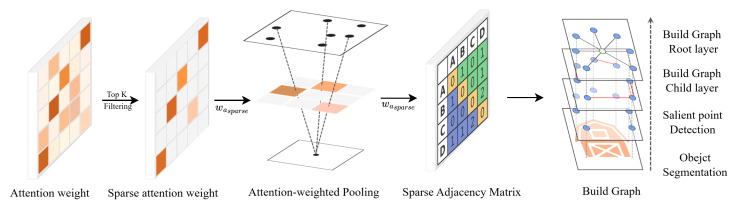
Process of center pooling and graph construction.

Instead of constructing a fully connected graph among all salient points, we apply a sparsely connected attention mechanism that selectively links only the most relevant pairs based on their importance. This approach helps reduce redundant computation and limits the risk of overfitting to local variations or noise. In the graph, directional edges are defined for both parent-to-child and child-to-child relationships, which allows the GNN to operate with fewer message-passing steps while maintaining a clear hierarchical information flow [59,60]. In our design, the object center is defined as the parent node, and salient points are treated as children. This hierarchical setup enables efficient message propagation by reducing redundant computation and enforcing structured information flow. To capture richer local context, additional child-to-child connections are selectively added based on attention weights, allowing semantically relevant features to interact without overburdening the graph. This dual-edge structure balances computational efficiency with representation expressiveness and was empirically found effective during experimentation. This process is illustrated in Figure 2, which visualizes the hierarchical graph structure consisting of the object center, directed edges to salient points, and selective child-to-child connections based on attention.

The resulting graph captures global structural context through the object center and preserves local geometric relations among the salient points. This compact yet informative structure forms the basis for the shape and color descriptors introduced in the following section.

#### 3.2.2. Shape Descriptor

Shape is a fundamental property of objects that tends to remain consistent under changes in lighting, texture, and viewpoint. To extract this geometric characteristic in a robust and generalizable manner, we propose a shape descriptor that integrates local features with a graph neural network (GNN), combined with a polar coordinate transformation.

The construction process begins with the graph nodes introduced in Section 3.2.1, which are transformed into a normalized coordinate space centered at the top-level central node. Each node’s position is converted into polar coordinates, and the relational structure is defined by the differences in radial distance and angular direction between nodes.

Compared to the Cartesian coordinate system, the polar coordinate system provides specific advantages for modeling object structure, particularly in cases where rotational invariance, radial symmetry, and center-based spatial organization are important [61,62]. These properties make it suitable for capturing object shape in a way that aligns with the spatial layout of real-world observations.

**Figure 3:**
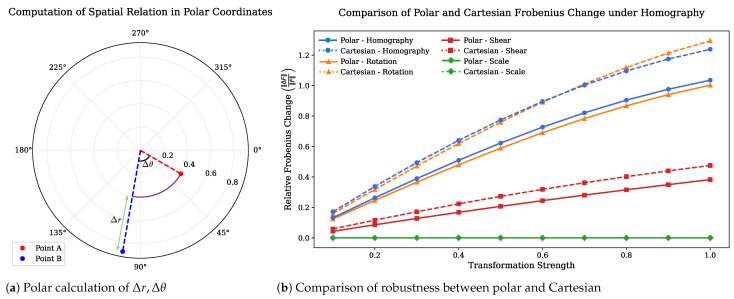
Comparison between (**a**) the calculation of Δr and Δθ in polar coordinates, and (**b**) the structural robustness of polar versus Cartesian coordinate representations.

In the polar coordinate system, the components of distance *r* and direction θ naturally separate, allowing rotational changes to be represented as simple shifts in the angular component while the radial value remains unchanged. This property is highly advantageous for object matching, symmetry analysis, and center-based pattern recognition, where robustness to orientation and rotation is essential. This directional information plays a critical role in representing the shape descriptor, more specifically, effectively capturing structural relations that are difficult to model using location-based representations alone. Figure 3 illustrates polar coordinates and its advantages. Figure 3a demonstrates how pairwise relationships between nodes compute in the polar coordinate system. Figure 3b shows the robustness of the proposed graph structure and polar coordinate to viewpoint changes commonly observed in natural images. It compares the variation in relational structures represented in Cartesian and polar coordinate systems under such transformations, quantified using the Frobenius norm [63].

**Figure 4:**
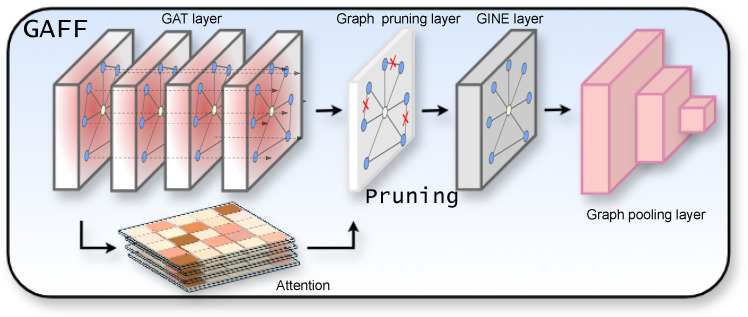
Graph filtering and fusion network.

We construct a sparse graph over these salient points using attention-based connectivity, where edges reflect learned local spatial relationships. The node features pass through a multi-layer graph neural network, aggregating information from neighboring salient points while preserving the overall structural layout. Constructing graphs based on image-derived features is inevitably vulnerable to structural distortions caused by false positives or false negatives. In particular, false positives can severely disrupt the overall graph structure by introducing spurious relationships between non-existent node pairs [57,64]. So, we introduce the GAFF (Graph Attention-based Feature Filtering) module, which selectively prunes structurally insignificant edges between GNN layers based on attention mechanisms. This module not only preserves the structural consistency but also the stability of the graph while enabling a more compact and robust representation. As a result, the proposed approach effectively learns reliable shape features under deformation and partial occlusion. The Figure 4 illustrates the structure of the GAFF module. The resulting shape embedding is a core component in our object representation.

#### 3.2.3. Color Descriptor

While shape captures the geometric structure of an object, color provides complementary information that is often essential for distinguishing between visually similar forms. Objects of the same category often exhibit similar shapes, and in such cases, color can serve as an important cue for distinguishing between objects [65]. However, color is highly susceptible to various external factors such as lighting, shadows, and imaging conditions, which can cause significant variations and hinder consistent representation. To address RGB color’s vulnerability, we propose a color descriptor that is robust to illumination changes while remaining effective for discriminating between different objects.

**Figure 5:**
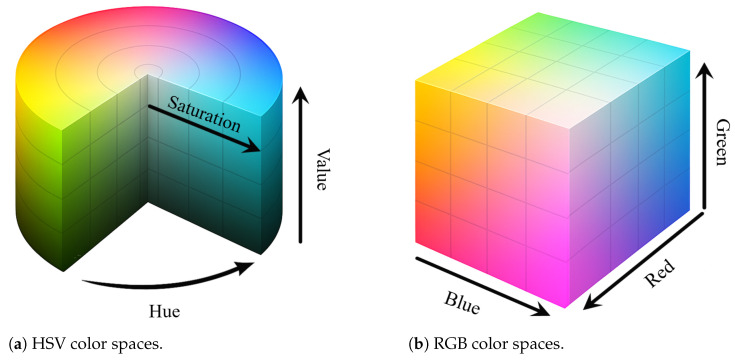
Comparison between (**a**) HSV and (**b**) RGB color spaces.

To encode visual appearance compactly and robustly, we design a color descriptor that uses normalized HSV color statistics. Unlike raw RGB values, the HSV color space decouples chromatic and intensity components, and it is more robust to lighting variations and shadows [66,67]. Most methods represent color using the RGB color space. However, RGB is not well aligned with human visual perception, particularly in capturing variations in hue. So, we adopt the HSV color space, which more effectively reflects perceptual color differences and better aligns with how humans perceive color. Figure 5 shows differences between the RGB and HSV color spaces and their respective approaches to color representation.

Unlike shape structures, color information is often less dependent on spatial position and can be more effectively and robustly represented through global distributions in color space, histogram-based statistics, or clustering methods [68]. Accordingly, in this study, we do not directly model color information within the graph structure. Instead, we represent only the presence or absence of relationships in the graph, while processing color features separately based on spatial continuity and statistical distribution characteristics. A learnable weighting function is applied over the color map based on the attention scores derived during the process described in Section 3.2.1, in order to emphasize relationships with high color contrast.

The color descriptors are subsequently integrated with shape and structural descriptors during the fusion stage, contributing to the effective discrimination of objects with similar appearances but differing material or color attributes. This fusion representation captures not only shape similarity but also reflects appearance differences, enabling more precise object distinction and recognition.

#### 3.2.4. Descriptor Fusion and Topology Embedding

Each descriptor module encodes a different aspect of the object representation: Shape is geometry, and Color is appearance. A fusion embedding is necessary for each informative descriptor in isolation to construct a complete and discriminative object representation. TOSD constructs a fusion descriptor that represents an object by fusing the shape descriptor and color descriptors. This encourages object discrimination without class information, and the resulting node embeddings demonstrate greater robustness compared to conventional off-the-shelf local features. This fusion approach aligns with recent multimodality feature extraction strategies [2], as it combines semantically distinct cues shape, color, and topology from a single modality to enhance object representation.

The topology descriptor is used to represent the overall scene by aggregating fusion descriptors and defining inter-object relationships in an object-centric manner. Although the construction method is related to the Shape Structure Graph introduced in Section 3.2.2, its purpose and implementation are different. Here, the graph does not focus on representing the internal structure of a single object but instead models the relationships between multiple objects. These relationships are defined using descriptor similarity, IoU, spatial distance, and semantic relevance, and are initially formed as a fully connected graph.

To reduce redundancy, irrelevant or weak connections are removed by the GAFF (Graph Attention-based Feature Filtering) module. As a result, only meaningful connections between major landmarks are preserved. This representation helps capture the semantic layout of the scene and allows efficient summarization of high-dimensional relational features.

As a result, the generated descriptor is based on a hierarchical structure that simultaneously captures low-level features at the pixel level, mid-level representations such as object shape and color, and high-level features encompassing the semantic content and spatial layout of the scene.Such an integrated representation enables simultaneous support for various visual tasks, including visual odometry, object tracking, global re-localization, and scene clustering. It also serves as a foundational component for the complete automation of our previous work, *Semantic Modeling Framework* [3,6].

### 3.3. Loss Functions and Training Objectives

To support hierarchical representation learning, we apply separate loss functions at each level of the hierarchy. In our framework, representations are structured across three levels: pixel, object, and scene. At each level, we use independent loss terms that correspond to specific features such as shape, color, semantics, and topology. These losses are formulated using contrastive learning, which encourages representations of anchor-positive pairs to be close within each descriptor space.

Beyond local descriptors, we also define a graph-level loss to capture the structural similarity across full objects or scenes. This additional loss operates on graphs and promotes similar embeddings for those with comparable edge patterns or connectivity structures. As a result, the method preserves high-level structure while improving the discrimination of individual representations.

The training data is constructed from images in the COCO [69] and SA-1B [7] datasets, where object-level segments are automatically extracted using FastSAM [8]. For each input image, we generate a transformed variant by applying visual modifications, including perspective distortion, brightness and contrast changes, and Gaussian blur. Based on the corresponding homography matrix and segmentation masks, matching object pairs are automatically detected and aligned between the original and transformed images.

From these aligned object pairs, salient points are extracted for each object. Descriptors are then learned by combining information from shape, color, semantic, and topological features. This procedure aims to train hierarchical representations that remain consistent and discriminative under changes in viewpoint and illumination.

#### 3.3.1. Low-Level Loss

Low-level training is designed to maximize the similarity between local descriptors in corresponding regions of image pairs, using given homography transformations. In this stage, shape and color information are integrated by combining node features obtained through GNN-based structural learning with the original local descriptors. This fusion results in a multimodal representation. Also, the center of each object is estimated in a robust manner, and salient points connected to this center are used to represent the object’s shape and color properties. This helps the representation learn these two aspects together in a consistent way.

To improve both the accuracy of the center estimation and the expression of shape and color, the convex hull formed by the selected salient points is encouraged to approximate the actual contour of the object. The salient points are not simply adjusted in position but are trained to be meaningful features that spread toward the object’s outer boundary while also reflecting the internal color distribution. This process leads to a structured form that encodes both geometric and appearance cues. During training, salient point detectors were either fixed or selectively fine-tuned depending on the objective, allowing the model to flexibly adapt feature selection to its learning goals.

(1) $$L_{\mathrm{low}}=\underset{\mathrm{shape}\;\mathrm{loss}}{\underset{︸}{1-\frac{\left|H\cap S\right|}{\left|H\cup S\right|}}}+\alpha \cdot \underset{\mathrm{color}\;\mathrm{distribution}\;\mathrm{divergence}}{\underset{︸}{D_{\mathrm{KL}}\left(P_{\mathrm{seg}}\;∥\;P_{\mathrm{salient}}\right)}}+\beta \cdot \underset{\mathrm{descriptor}\;\mathrm{similarity}\;\mathrm{loss}}{\underset{︸}{\frac{1}{N}\sum_{i=1}^{N}\left(1-\cos\left(f\left(p_{i}\right),f\left(q_{i}\right)\right)\right)}}$$

In Equation (1), the first term is a shape loss that the convex hull H formed by the salient points matches the segmentation mask S using an IoU-based shape loss. The second term is a color distribution loss that minimizes the Kullback–Leibler divergence [70] between the HSV color histogram $P_{\mathrm{seg}}$ of the object segment and $P_{\mathrm{salient}}$ from the salient point. This term encourages color-aware selection of salient points that reflect the overall color distribution of the object. The final term is a contrastive loss based on cosine similarity between matched salient point descriptors $\left(p_{i},q_{i}\right)$, ensuring that their feature representations remain consistent under geometric transformations. The weights α and β control the relative contribution of color and descriptor terms. The weights α=0.5 and β=0.9 were empirically selected as fixed values based on observed stability and overall performance across preliminary tests.

#### 3.3.2. Object-Level Loss

At the object level, each object is summarized into a single pooled vector, and contrastive learning is applied between these vectors to enable discriminative training across object instances.

(2) $$L_{\mathrm{obj}}=\underset{\mathrm{fusion}}{\underset{︸}{\mathrm{NTXent}(D_{a}^{\mathrm{fusion}},D_{p}^{\mathrm{fusion}})}}+\alpha \;\underset{\mathrm{shape}}{\underset{︸}{\mathrm{NTXent}(D_{a}^{\mathrm{shape}},D_{p}^{\mathrm{shape}})}}+\beta \;\underset{\mathrm{color}}{\underset{︸}{\left(1-\cos\left(D_{a}^{\mathrm{color}},D_{p}^{\mathrm{color}}\right)\right)}}$$

For the shape and fusion descriptors in Equation (1), we employ the NT-Xent [71]. Xent-loss encourages separate representations of different objects while encouraging consistent embeddings for the same object under various transformations, such as viewpoint and geometric distortions. In contrast, color representations are highly sensitive to variations in lighting, viewpoint, and surrounding environments, which can lead to discrepancies in color descriptors even for the same object. To account for this variability, a more flexible and soft loss formulation is required for the color branch. Therefore, instead of using a strong alignment objective such as NT-Xent, we adopt a soft contrastive approach that directly maximizes the cosine similarity between color descriptors. The weights α=1 and β=0.7 were empirically selected to balance the shape and color loss terms, while the remaining contribution was implicitly assigned to the descriptor similarity term. These fixed values were chosen based on empirical effectiveness across preliminary experiments.

#### 3.3.3. High-Level Loss

We define a high-level loss that creates a global embedding vector representing the overall scene in the image. Similar to the object-level loss, we apply a contrastive learning strategy to place semantically related scenes closer in the feature space. In addition to this, we include an edge pattern loss to reflect the structural similarity of scene-level graphs. This loss helps to maintain the morphological consistency of the graph and improves the stability of the representation.

Although methods such as GraphCL [69] focus on aligning global embeddings from different augmented versions of the same graph, they do not consider the detailed edge connectivity inside the graph. In our method, we add an explicit objective that aligns edge-level information to increase structural similarity between different graphs. To do this, we calculate the cosine similarity between the average edge feature vectors from each scene’s graph. Based on this, the edge pattern loss is defined, as shown in Equation (3).

(3) $$L_{\mathrm{edge}-\mathrm{pattern}}=1-\cos\left(\frac{1}{N_{a}}\sum_{i=1}^{N_{a}}e_{i}^{\left(a\right)},\frac{1}{N_{p}}\sum_{j=1}^{N_{p}}e_{j}^{\left(p\right)}\right)$$

Here, e denotes the edge feature vector, and *N* represents the total number of edges in the corresponding graph.

(4) $$L_{\mathrm{heigh}}=\underset{\mathrm{Topology}\;\mathrm{descriptor}}{\underset{︸}{\mathrm{NTXent}(D_{a}^{\mathrm{Topology}},D_{p}^{\mathrm{Topology}})}}+\lambda \cdot \underset{\mathrm{graph}\;\mathrm{similarity}}{\underset{︸}{L_{\mathrm{edge}-\mathrm{pattern}}}}$$

The final high-level loss is defined as shown in Equation (4). The weight λ=0.3 was empirically set to control the contribution of the graph similarity term. This fixed value was chosen to provide a moderate influence without overwhelming the primary descriptor losses.

## 4. Experiments

### 4.1. Datasets and Implementation Details

In this paper, we evaluate the performance of the proposed method across various vision tasks using usual benchmark datasets and a custom dataset. Specifically, HPatches [72] is used for image matching, OTB [73] and VOT2018 [74] for object tracking, KITTI [75] for visual odometry and re-localization, ROxford, and RParis [76] for image retrieval. These datasets are widely adopted benchmarks and include diverse scene variations and lighting conditions, making them suitable for assessing the generalization ability and robustness of the proposed model.

For object extraction, we utilize Fast Segment Anything [8], which can generate high-confidence object masks without additional training. This enables consistent object-level representations across diverse scenes. The extracted objects are processed automatically without requiring manual annotations, and each object’s salient points and descriptors are generated for subsequent processing. Notably, this zero-shot segmentation approach can also capture semantically unannotated yet structurally meaningful elements such as bridges, architectural decorations, or background structures, which are often excluded in class-based detectors. This broader coverage allows our framework to better represent the overall structure and context of a scene. SuperPoint [12] is employed for salient points extraction in this process. The model was trained using the COCO [69], SA-1B [7] dataset. Multiple loss components based on shape, color, and topology were employed, combined with equal weighting. The overall balance among the losses was empirically adjusted to optimize performance. All training and inference were conducted on an NVIDIA RTX 3090 GPU (Santa Clara, CA, USA).

### 4.2. Performance Evaluation

In the image matching experiments, we utilize off-the-shelf keypoint detectors such as SuperPoint to extract sparse local features. Based on these features, our network computes object-center and constructs a graph. In this process, salient points are represented as graph nodes, and within the structural context organized around the object center, the node features are naturally refined through message passing.

We evaluate feature matching performance using standard metrics, including *Mean Matching Accuracy (MMA)* and *Area Under the Curve (AUC)* of the cumulative matching accuracy curve.

- **MMA@r**: The percentage of keypoint matches with a reprojection error below threshold *r* pixels. We report MMA@3px in our results.
- **AUC@r**: The area under the cumulative curve of correct matches with respect to the reprojection error threshold, up to *r* pixels (e.g., AUC@2 and AUC@5).

**Figure 6:**
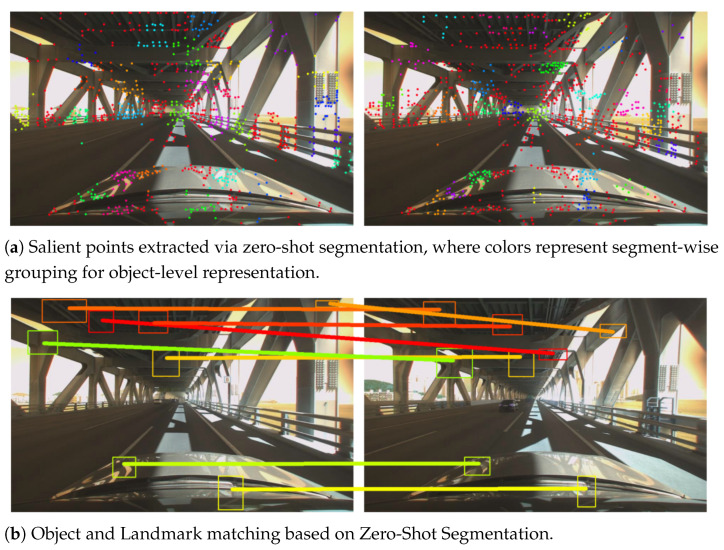
Object matching in real road environments: demonstrating the contribution of semantically unimportant objects to image representation.

As presented in Table 1, the proposed descriptor builds upon salient points extracted using SuperPoint in the preprocessing stage yet demonstrates greater robustness to illumination and viewpoint variations than raw SuperPoint features. This performance gain can be attributed to the effective use of salient point detection capability, followed by integrating structural and contextual information within an object-centric graph representation, enhancing the descriptor expressiveness. Overall, the proposed approach achieves competitive performance across various evaluation metrics, demonstrating the effectiveness of object-centric representation in the image matching task, as further illustrated in Figure 6 showing the contribution of semantically less important objects to robust matching in real road environments.

In the object tracking experiments, we verified that leveraging hierarchical descriptors effectively produces more precise object representation. We evaluated performance using three configurations: shape, color, and fusion. This allowed us to analyze the individual contributions of shape and color descriptors and observe that their combination yields complementary characteristics, resulting in improved overall robustness.

We evaluate tracking performance using standard metrics including *Success Rate (SR)*, *Precision Rate (PR)*, and *Expected Average Overlap (EAO)*. In addition, we report *Accuracy (A)* and *Robustness (R)* for VOT-style benchmarks.

- **Success Rate (SR)**: Proportion of frames in which the Intersection over Union (IoU) between predicted and ground truth bounding boxes exceeds a given threshold.
- **Precision Rate (PR)**: Percentage of frames in which the center distance between predicted and ground truth bounding boxes is below a defined threshold (typically 20 pixels).
- **Expected Average Overlap (EAO)**: Average IoU score over successful tracking segments.
- **Accuracy (A)**: Average overlap between predicted and ground truth.
- **Robustness (R)**: The number of tracking failures or reinitializations required.

**Figure 7:**
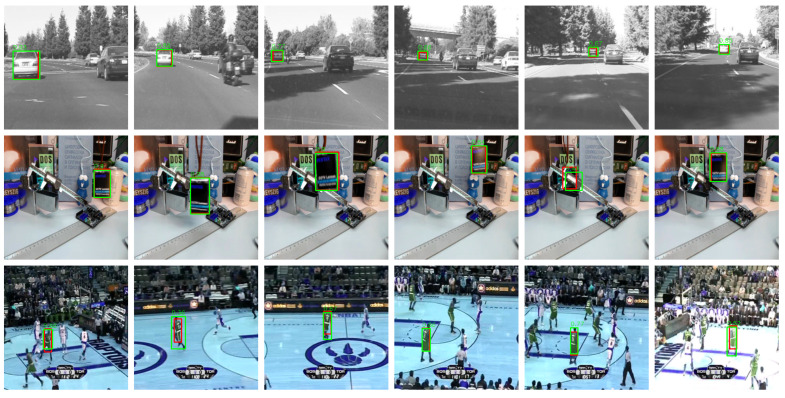
Result of object tracking across multiple frames.

When used alone, the shape descriptor effectively captures structural similarities between objects, while the color descriptor shows relatively lower tracking performance. However, when the two descriptors are fused, they provide meaningful support in distinguishing objects, as the complementary nature of shape and color leads to more stable tracking performance overall. Nonetheless, performance tends to degrade in small objects due to the limited number of extractable features. As shown in Table 2 and Figure 7, the proposed descriptor achieves performance comparable to or on par with existing methods across multiple evaluation metrics, demonstrating its practical potential for object tracking applications.

In this study, we perform a comparative experiment to analyze the impact of the proposed hierarchical representation approach on Visual Odometry, in contrast to conventional keypoint matching methods.

We evaluate Visual Odometry performance using the following standard metrics:

- **ATE (Absolute Trajectory Error)** [m]: The global consistency of the estimated trajectory by computing the root mean square error. (RMSE)
- **RPE (Relative Pose Error)** [m]: The local accuracy of the motion estimation by measuring the difference in relative pose over a fixed time interval.
- **Translation Drift** [%]: The accumulated translational error relative to the traveled distance.
- **Rotation Drift** [deg/m]: The average rotational error per meter traveled indicates the estimated motion’s angular stability.

**Figure 8:**
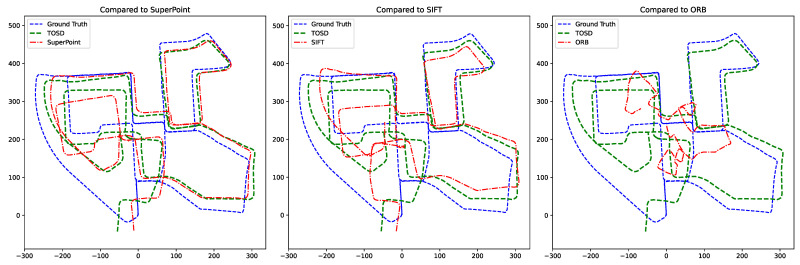
Qualitative trajectory comparison between the proposed method (TOSD) and conventional keypoint-based approaches.

The proposed method performs object-level matching and then aligns keypoints within the matched regions. That reduces erroneous correspondences caused by irrelevant background or dynamic objects while improving matching accuracy in structurally meaningful areas. However, due to the limitations of the object extraction stage, the total number of keypoints is reduced, which can lead to matching failures, particularly when important features are located in textureless regions or small objects. These results suggest that the object-centric representation effectively ensures structural consistency and semantic coherence; however, there is a trade-off in terms of keypoint density and processing speed. As shown in the performance comparison results in Table 3 and Figure 8, the proposed method outperforms conventional structural stability and matching accuracy approaches. However, there is a limit to what is affected by the number of keypoints and the computational efficiency.

To verify the applicability of the proposed descriptor in SLAM and localization tasks, we conducted a relocalization experiment based on the KITTI Odometry dataset. Image similarity is computed by combining object-level descriptors with global image-level features. In particular, the final matching score between two images is obtained by aggregating the similarities of corresponding object pairs that appear in both images. This approach enables more precise and meaningful similarity estimation compared to methods that rely solely on global feature averaging. A match is considered correct if the positional error is within 15 m and the orientation error is within 1 radian.

Performance is evaluated using three standard metrics: *Precision*, *Recall*, and *F1-score*, which collectively assess the accuracy, sensitivity, and balance of the image matching results.

- **Precision**: The proportion of predicted matches that are correct among all predicted matches.
- **Recall**: The proportion of correct matches that are successfully retrieved by the model among all ground-truth matches.
- **F1-score**: The harmonic mean of precision and recall, providing a balanced measure between the two.

As shown in Table 4, the proposed method outperforms the conventional global descriptor NetVLAD across all evaluation metrics. In particular, it demonstrates superior discriminative capability when matching structurally similar objects, compared to approaches based on simple global feature averaging. These results indicate that the object-centric representation enables more effective place re-identification even in complex urban environments.

**Figure 9:**
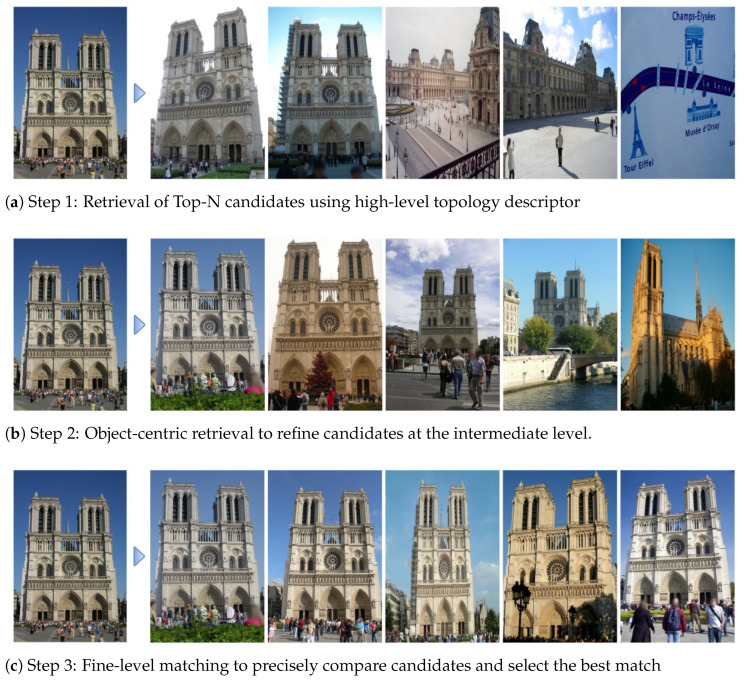
Overview of the hierarchical image retrieval pipeline.

This study aims to generate a global descriptor at the scene level by hierarchically representing images. To verify the effectiveness of the proposed descriptor, we conducted experiments to evaluate its applicability to image retrieval tasks. In these experiments, similarity-based filtering was performed from higher levels of the hierarchical structure, and the results confirmed that visually similar scenes could be effectively identified Figure 9.

We evaluate image retrieval performance on the ROxford and RParis benchmarks using the following standard metrics:

- **mAP (mean Average Precision)**: The mean of the average precision (AP) over all queries. AP is computed as the area under the precision-recall curve for each query. Higher mAP values indicate better retrieval performance.
- **Easy/Medium/Hard Protocols**: The benchmarks are evaluated under three difficulty levels based on viewpoint and appearance variations in the query-target pairs. Performance is reported separately for each level (E, M, H).

**Figure 10:**
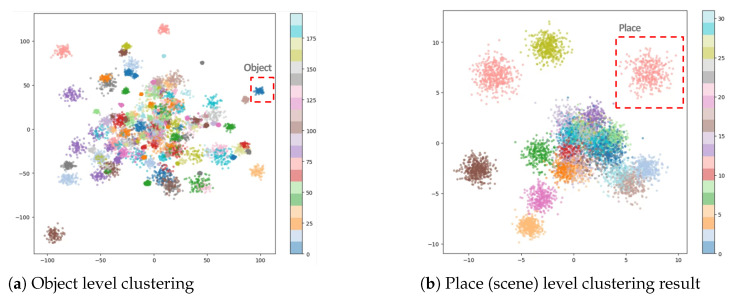
Clustering results of object-level and place-level grouping.

As shown in the results presented in Table 5, the proposed method demonstrated generally meaningful retrieval performance. In particular, it achieved competitive results compared to existing methods in cases where the scene structure was simple and the object composition was clear. However, compared to conventional image retrieval approaches that utilize rich local feature-based representations, our method exhibited limitations in more complex scenes with cluttered backgrounds or multiple overlapping objects. Nevertheless, the experimental results in Figure 10, confirm that the proposed descriptor provides sufficient discriminative power and practical performance for its intended application in scene-level image clustering.

In Table 5, “@” indicates the utilization of lower-level components, such as object matching or local feature matching.

### 4.3. Descriptor Contribution Analysis Under Appearance and Viewpoint Changes

To evaluate the individual contributions of shape and color descriptors and their fusion, we conduct a controlled experiment using the COCO dataset [69] with synthetic transformations. This setting simulates variations in viewpoint, illumination, and color tone by applying randomized geometric and photometric warping. Positive pairs are generated by applying different transformations to the same object, while negative pairs consist of semantically different but visually similar regions.

#### 4.3.1. Similarity Gap Analysis

We compute descriptor similarity scores between positive and negative pairs for shape, color, and fused descriptors. Table 6 summarizes the mean and standard deviation of the similarity scores, along with the gap between positive and negative means. A larger gap indicates a better discriminative capability.

#### 4.3.2. Object Matching

We further evaluate object matching performance using Recall@K and AUC. Table 7 presents the results. The fused descriptor achieves the best performance in all metrics, indicating that combining shape and color information yields more robust and discriminative representations.

These results confirm that the proposed fusion strategy successfully integrates geometric robustness and appearance sensitivity, resulting in improved performance under challenging viewpoint and lighting changes.

### 4.4. Computation Cost Analysis by Hierarchical Module

To assess the computational efficiency and structural clarity of our proposed hierarchical descriptor, we report both a model-level comparison and a module-wise breakdown, as presented in Table 8.

This analysis highlights the trade-offs between performance and computational demand, and it supports the modularity and interpretability of our design.

To further investigate the internal behavior of our hierarchical structure, we break down the inference time and parameter size by each semantic module.

As shown in Table 9, most of the inference cost is concentrated in the preprocessing (segmentation and local feature extraction) and the object-level descriptor pooling stages.

This breakdown helps identify computational bottlenecks and informs potential directions for runtime optimization.

## 5. Discussion

The experiment demonstrates that TOSD achieves competitive performance compared to baseline approaches in various vision tasks. Each descriptor plays a distinct and complementary role, and their fusion is essential for achieving high performance across diverse conditions.

The shape descriptor proved robust under viewpoint changes and occlusions due to its use of graph-based encoding and polar coordinate normalization. In particular, the attention-based selective unidirectional edge structure maintained accuracy while reducing computational cost. However, in cases where the object contour is not segmented or the distribution of salient points is sparse, shape-based embeddings may become unstable.

Color descriptor complements such cases by leveraging appearance cues, especially in environments with similar shape structures. Nevertheless, the reliability decreases in severe illumination changes, making integration with shape and topology information essential.

Topology descriptor provides a global context by modeling relationships between objects. This enables the identification of similar scenes and the execution of effective clustering based on topological structure. However, compared to recent methods, the limited feature representation led to performance degradation in precise tasks such as accurate image matching [18,52]. Additionally, multi-level hierarchical representations require relatively high computational resources. In addition to computational challenges, we also observed performance limitations in two specific scenarios: small objects and visually complex scenes. For small-scale objects, the segmentation model often fails to produce precise boundaries, resulting in an insufficient number of local features. This leads to sparse or incomplete graphs, ultimately degrading descriptor quality. In cluttered environments, dynamically changing elements such as vehicles or pedestrians may be extracted alongside static structures, disrupting the consistency of scene representation. These findings suggest that reliance solely on zero-shot segmentation may be suboptimal for such cases. As a remedy, future work will explore feature distillation from pretrained high-capacity networks and selective descriptor construction based on semantically meaningful object filtering, potentially leveraging image-text joint embedding models like CLIP to exclude noisy or irrelevant regions.

Furthermore, although TOSD is capable of extracting and integrating multi-level information in a unified framework, it does not always surpass conventional methods in task-specific benchmarks. This is partly because the method prioritizes structural and semantic richness over raw mathematical precision. Unlike traditional pipelines that optimize solely for pixel-wise accuracy, our framework emphasizes compositional and explainable scene understanding. By capturing object-level semantics and inter-object relations, TOSD enables generalizable representations that are less reliant on dataset-specific characteristics. In this way, the proposed architecture compensates for certain accuracy gaps through structured modeling and semantic flexibility, which are critical in open-world and multi-task environments.

However, even with these shortcomings, hierarchical representation is suitable for modeling the environment effectively. Also, low-level features can be utilized for visual odometry, mid-level features for defining key objects, and high-level features for scene clustering to define and recognize places. This makes the approach highly compatible with our previous work on the *Semantic Modeling Framework* [3].

## 6. Conclusions

This paper proposes a Hierarchical Object-centric Descriptor called TOSD (Triplet Object-Centric Semantic Descriptor) to overcome the limitations of conventional pixel-based and global embedding methods. TOSD describes hierarchical representations by integrating shape, color, and topological information without relying on class labels. This hierarchical representation provides multi-level semantic representations traversing pixels, objects, and scenes, enabling flexible integration of local details and global contextual information.

According to experiment results, TOSD has demonstrated rational performance in a range of visual odometry, SLAM, object tracking, global re-localization, scene clustering, and image retrieval. Importantly, TOSD does not rely on predefined class information, making it well-suited for unstructured environments and open-world scenarios. moreover, TOSD has the potential to serve as a fundamental technology for automating semantic-level visual understanding, owing to its strong compatibility with the Semantic Modeling Framework, which represents places, objects, and their ontological relationships using structured models. This integration positions TOSD as a crucial enabler for enhancing semantic cognition in both robotic and computer vision systems.

### Future Work

We will focus on enhancing real-time performance and integrating TOSD more practically into the *Semantic Modeling Framework*. To achieve this, we aim to move beyond the current off-the-shelf preprocessing approach by applying a descriptor distillation technique using high-performance models. This will allow us to extend the entire pipeline into an end-to-end framework. Moreover, to address the current limitations in handling small objects and cluttered backgrounds, we plan to replace segmentation-based object extraction with a feature-level abstraction strategy. In particular, we will investigate CLIP-style image–text joint embedding methods to filter and prioritize semantically meaningful regions, enabling more robust and context-aware object representations. Additionally, we aim to integrate multiscale feature representation to address the performance decline associated with differing object sizes. By extracting and combining features at various spatial resolutions, the proposed framework will effectively capture intricate details as well as broader contextual information, enhancing its robustness across a range of object scales.

## Figures and Tables

**Table 1:** Comparison with image matching on the Hpatch dataset. (↑: higher is better).

Methods	Hpatches			Methods	Hpatches		
	MMA@3 ↑	AUC@2 ↑	AUC@5 ↑		MMA@3 ↑	AUC@2 ↑	AUC@5 ↑
SIFT [9]	50.1	39.6	49.6	ASLFeat [77]	72.1	52.4	66.5
HardNet [78]	62.1	42.6	59.9	LoFTR [15]	81.2	58.7	74.5
DELF [53]	56.7	41.0	54.2	Key.Net [79]	73.2	54.0	68.5
LLF [80]	56.2	41.2	50.4	ALIKE [81]	70.5	51.7	66.9
Lf-net [82]	53.2	38.7	48.7	MTLDesc [83]	77.6	56.5	71.4
ContextDesc [84]	63.3	44.6	59.0	PoSFeat [85]	76.9	56.0	69.9
DISK [86]	72.2	52.3	66.4	SFD2 [87]	77.8	56.1	70.6
R2D2 [14]	64.4	45.8	61.6	TPR [88]	79.8	57.1	73.0
D2Net [13]	40.3	31.6	39.5	SuperPoint [12]	63.0	44.1	59.6
TOSD (shape)	66.3	49.4	64.1	TODS (fusion)	71.1	52.7	65.3

**Table 2:** Comparison with object tracking on OTB and VOT2018 benchmarks. (↑: higher is better, ↓: lower is better).

Tracker	OTB50		OTB100		Tracker	VOT2018		
	SR ↑	PR ↑	SR ↑	PR ↑		EAO ↑	A ↑	R ↓
SRDCF [89]	0.726	0.81	0.605	0.729	GFS-DCF [90]	0.397	0.511	0.143
LCT [91]	0.711	0.780	0.61	0.655	ATOM [92]	0.401	0.590	0.204
Staple [93]	0.745	0.766	0.593	0.848	SiamBAN [94]	0.452	0.597	0.178
HDT [95]	0.603	0.889	0.539	0.848	KYS [96]	0.446	0.598	0.191
ADNet [97]	0.659	0.903	0.590	0.803	KeepTrack [98]	0.476	0.615	0.172
CSR-DCF [99]	0.678	0.773	0.587	0.733	DTT [100]	0.449	0.615	0.176
SiamRPN [101]	0.663	0.800	0.631	0.853	TransT [102]	0.447	0.616	0.201
DCFNet [103]	0.618	0.716	0.618	0.804	SiamFC++ [104]	0.426	0.587	0.183
TOSD (shape)	0.617	0.767	0.562	0.767	TOSD (shape)	0.351	0.515	0.166
TOSD (color)	0.501	0.708	0.443	0.664	TOSD (color)	0.307	0.410	0.147
TOSD (fusion)	0.678	0.836	0.600	0.781	TOSD (fusion)	0.391	0.546	0.191

**Table 3:** Comparison with visual odometry on the KITTI dataset. (↓: lower is better).

Method	ATE (m) ↓	RPE (m) ↓	Trans. Drift (%) ↓	Rot. Drift (deg/m) ↓
Sift	158.9	0.385	9.63	0.549
ORB	392.2	0.31	10.5	1.08
Superpoint	338.8	0.262	9.1	0.82
TOSD	156.5	0.263	4.2	0.778

**Table 4:** Comparison with re-localization on the KITTI dataset. (↑: higher is better).

Sequence	Method	Precision ↑	Recall ↑	F1 ↑	Method	Precision ↑	Recall ↑	F1 ↑
00	NetVLAD [52]	0.211	0.413	0.279	TOSD	0.799	0.578	0.638
05	NetVLAD [52]	0.190	0.253	0.217	TOSD	0.720	0.399	0.490
06	NetVLAD [52]	0.390	0.274	0.322	TOSD	0.705	0.441	0.452

**Table 5:** Comparison with traditional global descriptors on ROxford and RParis benchmarks.

Method	Dimensions	Roxford			RParis		
		Easy	Medium	Hard	Easy	Medium	Hard
V-[O]-MAC [76]	512	0.587	0.446	0.198	0.592	0.359	0.176
V-[O]-SPoC [76]	512	0.601	0.459	0.212	0.598	0.324	0.158
V-[O]-CroW [76]	512	0.612	0.472	0.225	0.629	0.369	0.184
V-[O]-GeM [76]	512	0.623	0.483	0.237	0.632	0.388	0.196
V-[O]-R-MAC [76]	512	0.635	0.496	0.248	0.662	0.409	0.208
TOSD (topology)	512+@	0.620	0.449	0.185	0.624	0.324	0.147
TOSD (topology)	256+@	0.591	0.429	0.183	0.594	0.319	0.142

**Table 6:** Descriptor similarity on warped COCO.

Descriptor	Mean (Pos)	Std (Pos)	Mean (Neg)	Std (Neg)	Gap (Pos − Neg)
Shape	0.815	0.061	0.335	0.063	0.752
Color	0.783	0.079	0.369	0.071	0.712
Fusion	**0.852**	**0.043**	**0.270**	**0.051**	**0.801**

**Table 7:** Object matching performance on warped COCO.

Descriptor	Recall@1	Recall@5	Recall@10	AUC
Shape	0.571	0.598	0.633	0.699
Color	0.470	0.487	0.522	0.646
Fusion	**0.631**	**0.662**	**0.693**	**0.735**

**Table 8:** Comparison of parameter size and inference speed across various visual descriptors.

Model	Parameters (M)	FPS	Description
SuperPoint + SuperGlue [12,105]	13.30	24.28	Sparse keypoint detection and matching
LoFTR [15]	11.56	8.32	Dense feature-level matching
SAM [7]	641.09	2.10	Object-level zero-shot segmentation
DINOv2-base [56]	86.58	122.06	Patch-level ViT feature embedding
DINOv2-giant [56]	1136.48	22.79	Large-scale scene transformer
DETR [106]	43.04	18.48	End-to-end object detection
**Ours**	**25.37**	**12.70**	Hierarchical Object-Centric Descriptor

**Table 9:** Module-wise breakdown of our descriptor in terms of parameters and inference time.

Module	Parameters (M)	Time (ms)	Description
Preprocess	13.10	34.10	Segmentation and keypoint detection
Low-level	6.73	2.85	Salient point abstraction and local encoding
Object-level	3.56	35.20	Object-wise pooling and relational encoding
Scene-level	1.98	6.59	Scene-level aggregation via object graph
**Total**	**25.37**	**78.74**	Full hierarchical descriptor

## Data Availability

The data presented in this study are not publicly available due to project confidentiality agreements. Further inquiries can be directed to the corresponding author.
